# Multislice computerized tomography coronary angiography can be a comparable tool to intravascular ultrasound in evaluating “true” coronary artery bifurcations

**DOI:** 10.3389/fcvm.2023.1292517

**Published:** 2023-11-06

**Authors:** Anja Radunović, Radosav Vidaković, Stefan Timčić, Natalija Odanović, Milica Stefanović, Mirko Lipovac, Kosta Krupniković, Aleksandar Mandić, Dejan Kojić, Milosav Tomović, Ivan Ilić

**Affiliations:** ^1^ Department of Cardiology, Institute for Cardiovascular Diseases Dedinje, Belgrade, Serbia; ^2^ Department of Cardiology, Clinical Hospital Center Zemun, Belgrade, Serbia; ^3^ Faculty of Medicine, University of Belgrade, Belgrade, Serbia

**Keywords:** CT coronary angiography, PCI, IVUS, coronary artery bifurcation, atherosclerosis plaque

## Abstract

**Aim:**

Coronary bifurcation atherosclerosis depends on its angles, flow, and extensive branching. We investigate the ability of CT coronary angiography (CTCA) to determine atherosclerotic plaque characteristics of “true” bifurcation compared with intravascular ultrasound (IVUS) and the influence on side branch (SB) fate after percutaneous coronary intervention (PCI).

**Methods and results:**

The study included 70 patients with 72 “true” bifurcations. Most of the bifurcations were in the left anterior descending—diagonal (Dg) territory [50 out of 72 (69.4%)]. Longitudinal plaque evaluation at the polygon of confluence [carina and 5 mm proximal and distal in the main branch (MB)] showed that carina side MB and SB plaque had occurred with the lowest incidence with fibro-lipid structure (115 ± 63 HU and 89 ± 73 HU, *p* < 0.001 for all). Bland–Altman analysis showed a discrepancy in measuring mainly the lumen area between CTCA and IVUS in proximal MB [lumen 5.10, 95% CI (95% confidence interval, 4.53–5.68) mm^2^, *p* < 0.001; vessel −1.42, 95% CI (−2.63 to −0.21) mm^2^, *p* = 0.023], carina MB [lumen 3.74, 95% CI (3.37–4.10) mm^2^, *p* < 0.001; vessel −0.48, 95% CI (−1.45 to 0.48) mm^2^, *p* = 0.322], and distal MB [lumen 4.72, 95% CI (4.27–5.18) mm^2^, *p* < 0.001; vessel 0.62, 95% CI (−0.53 to 1.77) mm^2^, *p* = 0.283]. A significant correlation existed between average plaque density on CTCA with a percentage of calcified plaque on IVUS tissue characterization (proximal *r* = 0.307/*p* = 0.024, carina 0.469/0.008, distal 0.339/0.024, minimal lumen diameter 0.318/0.020). Circumferential plaque in the proximal MB segment remained an independent predictor of SB compromise [OR 3.962 (95% CI 1.170–13.418)].

**Conclusion:**

Detection and characterization of atherosclerotic plaque by CTCA in non-left main “true” coronary bifurcations can provide useful information about bifurcation anatomy and plaque distribution that can predict outcomes after provisional stenting, thus guiding the interventional strategy to bifurcation PCI.

## 1. Introduction

Coronary artery disease (CAD) is still one of the leading causes of mortality in developed counties, and approximately 7 million people in the world every year have a confirmed diagnosis of acute coronary syndrome (ACS) (1). Percutaneous coronary interventions (PCI) in bifurcation lesions comprise approximately 15%–20% of all PCI procedures and are associated with an increased incidence of restenosis and stent thrombosis (2, 3). No single PCI technique that is universally applicable to all patients with coronary bifurcation lesions is available, but the preferred strategy for most of them, depending on distribution of atherosclerosis in main branch (MB) and side branch (SB), would be first to stent the MB across SB (4). Treatment of “true” bifurcation lesions, which have a significant stenosis of more than 50% in both MB and SB, especially if the plaque extends distally from the SB ostium, remains challenging, also often requiring stent implantation in the SB (5).

Multislice computerized tomography coronary angiography (CTCA) has become an important tool for establishing the diagnosis of ischemic heart disease, allowing detection and tissue characterization of coronary artery atherosclerotic lesions (6, 7). CTCA has been extensively explored as a non-invasive method that can provide useful information regarding plaque distribution and characteristics that can be helpful in planning and execution of complex PCI. CTCA plays a major role in PCI guidance of chronic total occlusion (CTO) that enables evaluation of occlusion length and distribution and composition of the atherosclerotic plaque (8–10). CTCA enables non-invasive evaluation of three-dimensional geometry of coronary bifurcations, including angle measurement and plaque detection. It provides complete visualization of angulation of the coronary tree preventing foreshortening and overlapping of bifurcation segments that is common in conventional angiography (11, 12). Based on the CTCA, several scoring systems were developed that served to predict the outcomes of bifurcation PCI, such as V-RESOLVE (Risk prEdiction of Side branch OccLusion in coronary bifurcation interVEntion) score (13). In comparison with intravascular ultrasound (IVUS), CTCA showed a good correlation in identification and quantification of intermediate atherosclerotic lesions (14, 15).

Therefore, we investigated whether the characteristics of atherosclerotic plaque in MB and SB in “true” non-left main bifurcation lesions, determined by CTCA, can influence the occurrence of SB compromise after MB provisional stenting and to compare bifurcation plaque analysis between CTCA and IVUS.

## 2. Materials and methods

The study was prospective and conducted in a PCI center of a single, high-volume tertiary university. It included patients with “true” native coronary artery bifurcations (Medina 1.0.1; 0.1.1; 1.1.1) with a visually estimated stenosis of >50% in both MB and SB, with an SB diameter greater than 2 mm. Patients were scheduled to undergo PCI based on the history of typical stable angina, silent ischemia, and/or proven ischemia on functional testing. The study was approved by the institutional ethics committee and was completed in accordance with the Helsinki Declaration, and it was a part of the project that evaluated the use of multimodality imaging with CTCA, IVUS, and invasive coronary angiography in “true” coronary bifurcations treated with PCI according to recent guidelines (the study protocol was registered at www.clinicaltrials.gov—NCT 01943643) (4).

### 2.1. Study population

The study included patients with chronic coronary syndrome; therefore, patients with ACS were not included. Moreover, patients were not considered for the study if they have left ventricular ejection fraction (LVEF) of less than 30% or suffering from renal failure with estimated glomerular filtration rate (eGFR) of less than 30 ml/min/m^2^. Patients with bifurcation lesion within the culprit artery causing myocardial infarction, grafted surgically or previously treated with PCI, were not considered for the study. Patients having medical conditions that can be a contraindication for CTCA and/or PCI were also excluded from the study. Due to planned CTCA investigation and complex PCI, we felt that it would be unethical to perform calcium scoring prior to CTCA. However, based on CTCA findings, patients with extensive calcifications at the site of bifurcation planned to be treated (with circumferential calcification greater than 180° and/or 5 mm or more in length) were excluded.

### 2.2. CTCA procedure

**Figure 1:**
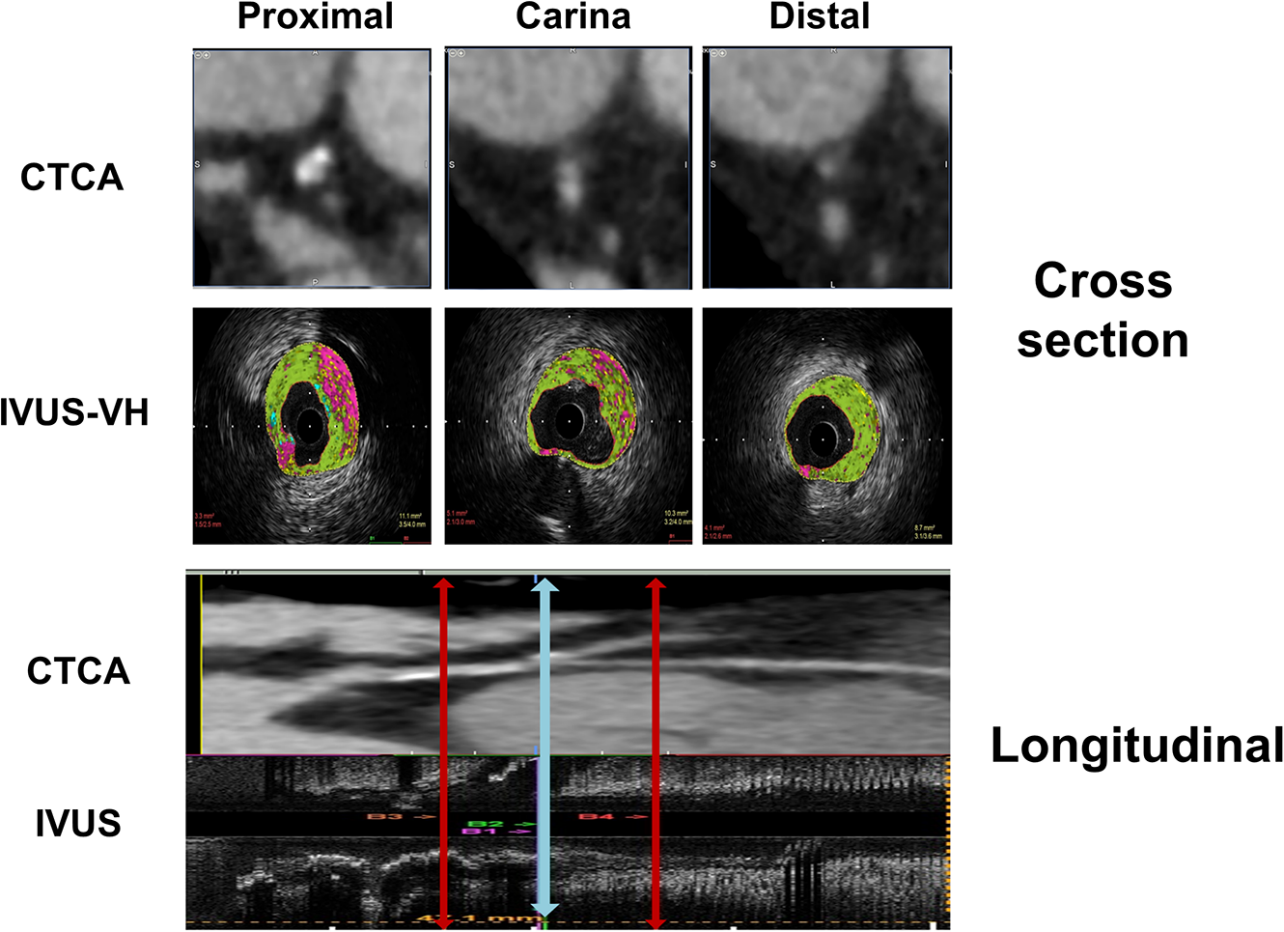
Diagram represents the CT coronary angiography and IVUS longitudinal and cross-sectional view of the bifurcation with segments included in quantitative and plaque analysis.

**Figure 2:**
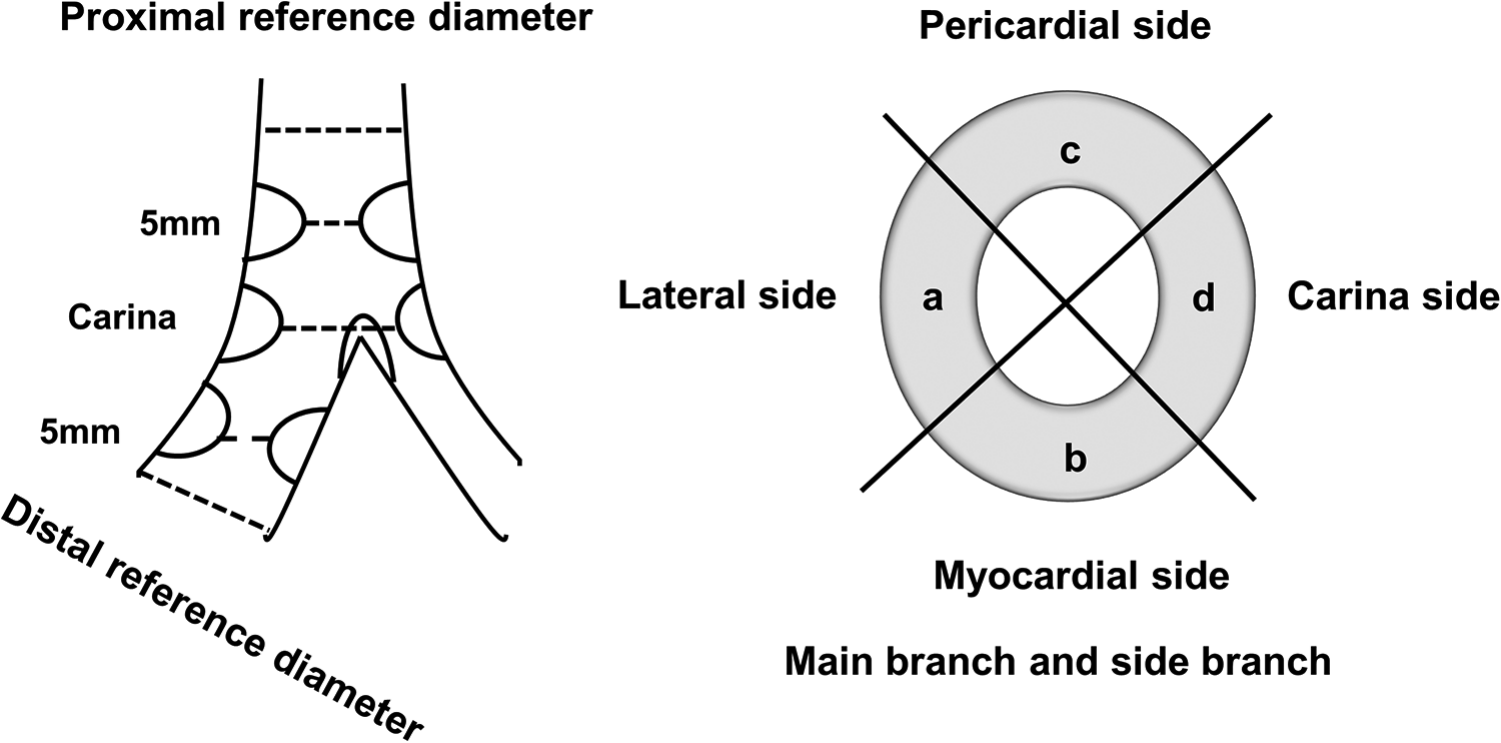
Longitudinal and cross-sectional views of the bifurcation with segments included in analyses of CTCA diameters and plaque characterization.

Patients underwent CTCA on Aquilion CXL 128 slice CT scanner (Toshiba Medical Systems Europe, Zoetermeer, Netherlands) using a predefined protocol and Ultravist 370 contrast agent (with an iopromide concentration of 370 mg/ml, Bayer Health Care, Germany). The angiograms were analyzed offline using dedicated software Vital Vitrea Advanced 6.2 (Vital Images, Minnetonka, MN, USA). The bifurcation lesion analysis included the measurement of proximal and distal reference vessel diameters (RVD) of the MB and RVD of the SB at the point least affected by atherosclerosis of up to 10 mm from the bifurcation's stenosis; MB vessel and lumen diameters and areas 5 mm proximal to the carina, at the carina level, and 5 mm distal to the carina (polygon of confluence); and at the site of MB's greatest luminal narrowing—minimal lumen diameter (MLD). The same analysis was performed at the SB ostium. Moreover, the angles between MB and SB were measured. The plaque analysis was performed in the longitudinal cross-section of the bifurcation at the lateral and carina sides at every previously mentioned location in the MB and SB (Figure 1). Circumferential cross-sectional plaque analysis was performed at the points of “polygon of confluence” to locate the presence of circumferential plaque. The plaques were identified at the pericardial, myocardial, lateral, and medial sides of the vessel. Circumferential plaque was defined as the presence of atherosclerotic plaque at every side of the vessel at the predefined points in “polygon of confluence” and at the ostium of the SB (Figure 2). The plaque analysis in the longitudinal cross-section included determination of the type of the tissue based on a measured density in Hounsfield units (HU), so that the plaque with a density of up to 75 HU was marked as lipid, 76–130 HU as fibro-lipid, 131–350 HU as fibrous, and above 351 HU as calcified plaque (16).

A detailed description of CTCA procedure is available in Supplementary Data File.

### 2.3. IVUS procedure

After placing coronary guidewires in MB and SB, MB IVUS was performed using an Opticross 40 MHz IVUS catheter (Boston Scientific Corporation/SciMed, Natick, MA, USA). The withdrawal of the catheter was performed using an automated motorized pullback at a constant speed of 0.5 mm/s. The acquired data were stored and transferred for an offline analysis using commercially available software QIvus 3.0 (Medis Medical Images Software, Leiden, Netherlands). The lumen and the media–adventitia borders were defined by automatic contour detection software with manual editing at the frames of interest. Tissue characterization was done using iMAP software (Boston Scientific Corporation/SciMed, Natick, MA, USA) which uses color codes to describe the composition of the atherosclerotic plaque so that the fibrous tissue is coded green, lipid as yellow, necrotic as purple, and calcified plaque as light blue. The results were expressed as the percentage of the total segmental plaque for each tissue.

The anatomical co-registration between two imaging modalities was performed by identifying the carina on IVUS longitudinal and cross-section as a last frame where the entire circumference of MB and SB can be visualized before confluence. The points 5 mm proximal and distal to the carina in “polygon of confluence” were then identified. On longitudinal IVUS recording, the distance of the MLD point from the bifurcation's carina was calculated. The carina was identified on CTCA cross-section using the same principle as in IVUS recording, and then other three points of interest were detected using distances obtained in the IVUS longitudinal section (Figure 1).

### 2.4. PCI procedure

An initial strategy for PCI in all cases was “provisional” stenting of the MB. The choice of vascular access, guiding catheters, and coronary wires were left to the operators’ discretion. After MB predilation, a second-generation drug-eluting stent (DES) was placed across the SB, with a diameter chosen according to the visual estimate of the distal MB in accordance with Murray's law (4). After performing the proximal optimization technique (POT) using a short non-compliant balloon catheter, coronary angiograms in two orthogonal projections were performed. If the SB coronary blood flow was less than thrombolysis in myocardial infarction (TIMI) 3, the procedure was continued with angioplasty of the SB ostium. If after angioplasty and/or kissing balloon inflation the SB TIMI flow was less than 3 and/or there was an ostial dissection, another second-generation DES was implanted in the SB using the technique chosen by the operator. A detailed description of the PCI procedure and postprocedural treatment is available in Supplementary Data File.

### 2.5. Statistical analysis

Continuous data were summarized as the means ± SD. Categorical data were summarized as counts and percentages. Unpaired *t*-test was used for comparing the continuous variables, and the chi-squared test and Fisher's exact test were used for categorical variables. The effect of the patient's characteristics on the endpoint of SB compromise, defined as a decrease in flow below TIMI 3, was assessed using a logistic multivariable model, and the impact of these covariates was expressed as odds ratios with 95% confidence intervals (95% CI). A set of variables was prospectively selected, and a stepwise selection process was used to determine independent predictors of SB compromise in a multivariate analysis model. The correlation between CTCA and IVUS was assessed using Pearson's linear correlation. The mean differences and limits of agreement between these two imaging modalities were assessed by Bland–Altman analysis. The 95% limits of agreement were defined as the range of values within ± 2 standard deviations (SD) from the mean difference. A *p*-value of <0.05 was considered statistically significant. All statistical analyses were performed using PASW Statistics 18.0 statistical software (SPSS Inc., Chicago, IL, USA).

## 3. Results

Initially 123 patients were prospectively evaluated for inclusion in the study. Eight patients denied informed consent to participate in the study, 26 patients had extensive calcifications that precluded plaque analysis, 14 patients' angiograms did not fulfill the criteria for “true” bifurcation on CTCA due to insignificant stenosis of any of the bifurcation's branches, and five patients had angiograms not of suitable quality for interpretation.

### 3.1. Clinical and PCI results

The study included 70 patients with 72 “true” non-left main bifurcations. Their mean age was 60.2 ± 9.4 years, and the 71.4% of patients were males. Patients included in the study were found to have high incidence of hypertension, dyslipidemia, previous myocardial infarction, and previous PCI (Table 1).

**Table 1:** Clinical characteristics of the patients.

Variable	*n* = 70
Age (years)	60.2 ± 9.4
Male gender (%)	71.4
Heredity (%)	44.2
Smoking (%)	42.0
Hypertension (%)	92.7
Dyslipidemia (%)	72.4
Diabetes mellitus (%)	18.8
Insulin-dependent DM (%)	5.9
PAD (%)	5.8
Previous MI	55.1
Previous CVA (%)	2.9
Previous PCI (%)	55.0
Previous CABG (%)	0.0
BMI (kg/m^2^)	28.4 ± 3.9
LVEF (%)	51 ± 11
eGFR (ml/min/m^2^)	85.6 ± 22.7

BMI, body mass index; CABG, coronary artery bypass grafting; CVA, cerebrovascular event; DM, diabetes mellitus; eGFR, estimated glomerular filtration rate; LVEF, left ventricular ejection fraction; PAD, peripheral arterial disease; PCI, percutaneous coronary intervention.

Most of the patients included in the study had two-vessel disease [one-vessel disease, 17 out of 70 (24.3%); two-vessel disease, 43 out of 70 (61.4%); and three-vessel disease, 10 out of 70 (14.3%)]. Most of the bifurcations were in the left anterior descending (LAD)—diagonal (Dg) territory. All interventions were deemed successful by the operator with final TIMI 3 flows in MB and SB. Decreased coronary flow TIMI < 3 occurred in 17 out of 72 bifurcations (23.6%). It was treated by either balloon angioplasty only of the SB ostium [POT-side-POT, nine out of 17 (52.9%)] or stent implantation [eight out of 17 (47.1%)] followed by kissing balloon inflation and repeated POT (Table 2). During the CTCA procedure, the patients were exposed to dose-length product (DLP) of 414.18 ± 245.39 mGy cm, which was equivalent to exposure dose of 5.79 ± 3.43 mSv. The fluoroscopy time for the intervention was 48 ± 35 min, the amount of contrast used was 143 ± 57 ml, and patients’ radiation exposure was 1.02 ± 0.34 Gy.

**Table 2:** Angiographic and PCI characteristics of treated bifurcations.

Bifurcation characteristics	*n* = 72
Location *n* (%)	
LAD-Dg	50 (69.4)
Cx-OM	17 (23.6)
RCA PD-PL	5 (6.9)
Medina classification *n* (%)	
1.0.1	16 (22.2)
0.1.1	25 (34.7)
1.1.1	31 (43.0)
RVD MB (mm)	3.6 ± 0.4
RVD SB (mm)	2.6 ± 0.5
Stent diameter in MB (mm)	3.1 ± 0.4
Stent length in MB (mm)	25.8 ± 5.8
Maximum stent inflation (atm)	14.0 ± 1.4
SB TIMI < 3 *n* (%)	17 (23.6)
POT-side-POT *n* (%)	9 (52.9)
Stent in SB *n* (%)	8 (47.1)

Cx, circumflex; Dg, diagonal; OM, obtuse marginal; PD, posterior descending; PL, posterolateral; POT, proximal optimization technique; RCA, right coronary artery; RVD, reference vessel diameter.

### 3.2. CTCA results

**Figure 3:**
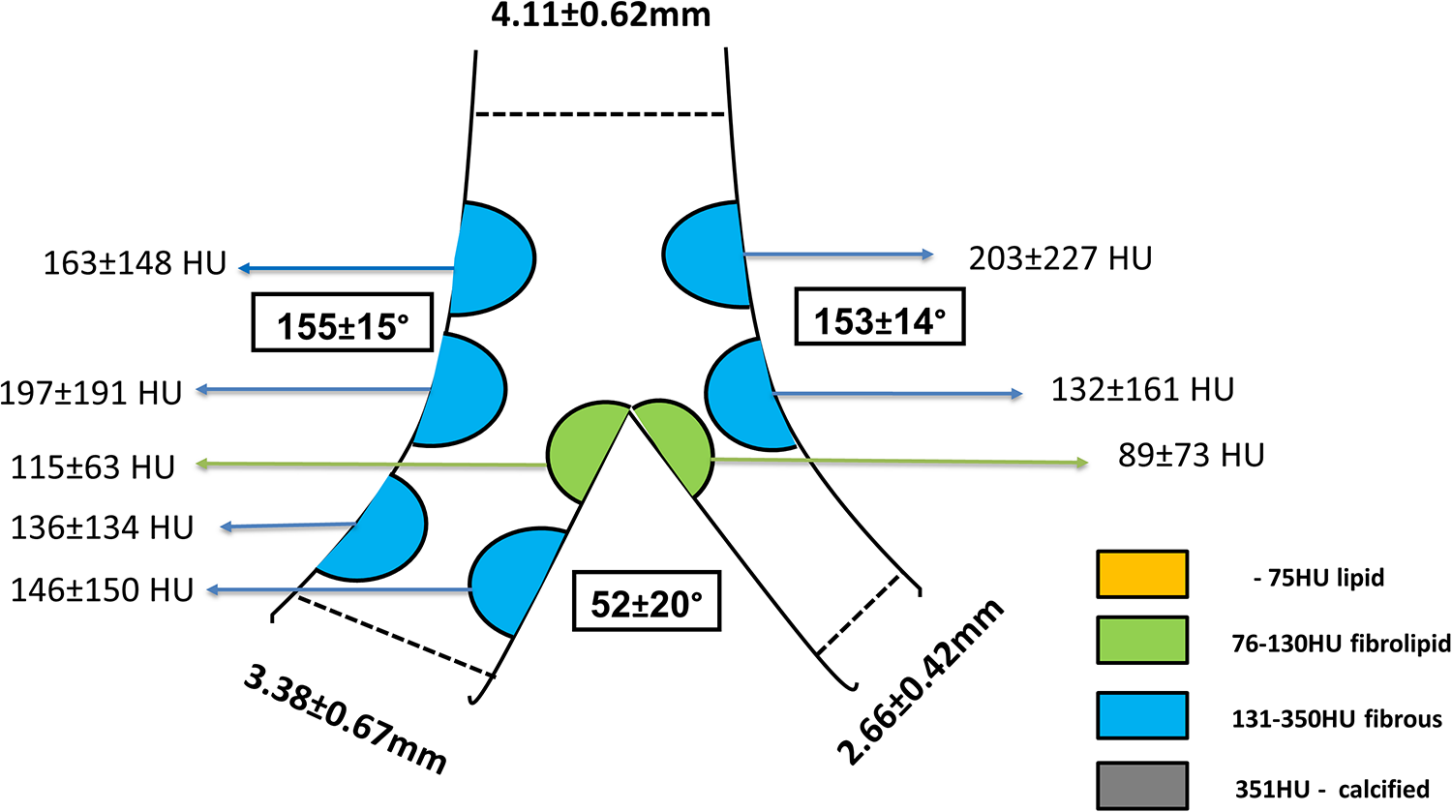
Longitudinal view of the bifurcation including bifurcation angles and plaque distribution and densities. Plaques were colored according to their densities in Hounsfield units (HU) and grouped into categories displayed in the legend.

On CTCA, the data acquired at longitudinal cross-section of bifurcation were presented in Figure 3, including bifurcation angles with plaque densities. The average reference vessel diameters were as follows: the MB reference diameter was 3.55 ± 0.67 mm and SB ostium 2.66 ± 0.42 mm, while the MB/SB ratio was 1.3 ± 0.3. Plaques on the carina side of the bifurcation's MB and SB carina fibro-lipid were compared with the fibrous ones on the lateral side of both MB (197 ± 191 HU vs. 115 ± 63 HU, *p* = 0.001) and SB (132 ± 161 vs. 89 ± 73 HU, *p* = 0.01). In the cross-sectional view, circumferential plaque occurred most frequently at the MLD level (39 out of 72 bifurcations, 54%), while in other measured segments, its incidence was lower: proximal MB (27 out of 72, 37%), carina-level MB (26 out of 72, 36%), distal MB (21 out of 72, 29%), and SB ostium (14 out of 72, 19%). In a multivariate regression analysis that included previously recognized predictors of SB compromise (diabetes, bifurcation angle, plaque at the carina side of MB and SB, circumferential plaque proximal and at the level of carina in the MB, circumferential plaque at SB ostium, ratio of MB/SB diameters), only proximal MB circumferential atherosclerotic plaque remained an independent predictor of decreased SB flow after stent implantation [3.962 (95% CI 1.170–13.418), *p* = 0.027] (Table 3).

**Table 3:** Univariate and multivariate predictors of SB compromise (TIMI < 3).

	Univariate		Multivariate	
	OR (95% CI)	*p*-value	OR (95% CI)	*p*-Value
Presence of diabetes	1.333 (0.355–5.011)	0.670	—	—
Bifurcation angle	1.041 (0.995–1.090)	0.085	1.041 (0.995–1.090)	0.084
MB/SB reference diameters	0.794 (0.082–7.711)	0.842	—	—
SB carina plaque in longitudinal view	1.307 (0.413–4.133)	0.649	—	—
MB carina plaque in longitudinal view	0.891 (0.289–2.748)	0.840	—	—
Circumferential plaque at MB carina	1.450 (0.477–4.409)	0.513	—	—
Circumferential plaque at proximal MB	3.480 (1.114–10.864)	0.032	3.962 (1.170–13.418)	0.027
Circumferential plaque at SB ostium	3.250 (0.883–11.967)	0.076	1.432 (0.450–4.561)	0.543

### 3.3. Quantitative results of CTCA and IVUS

**Figure 4:**
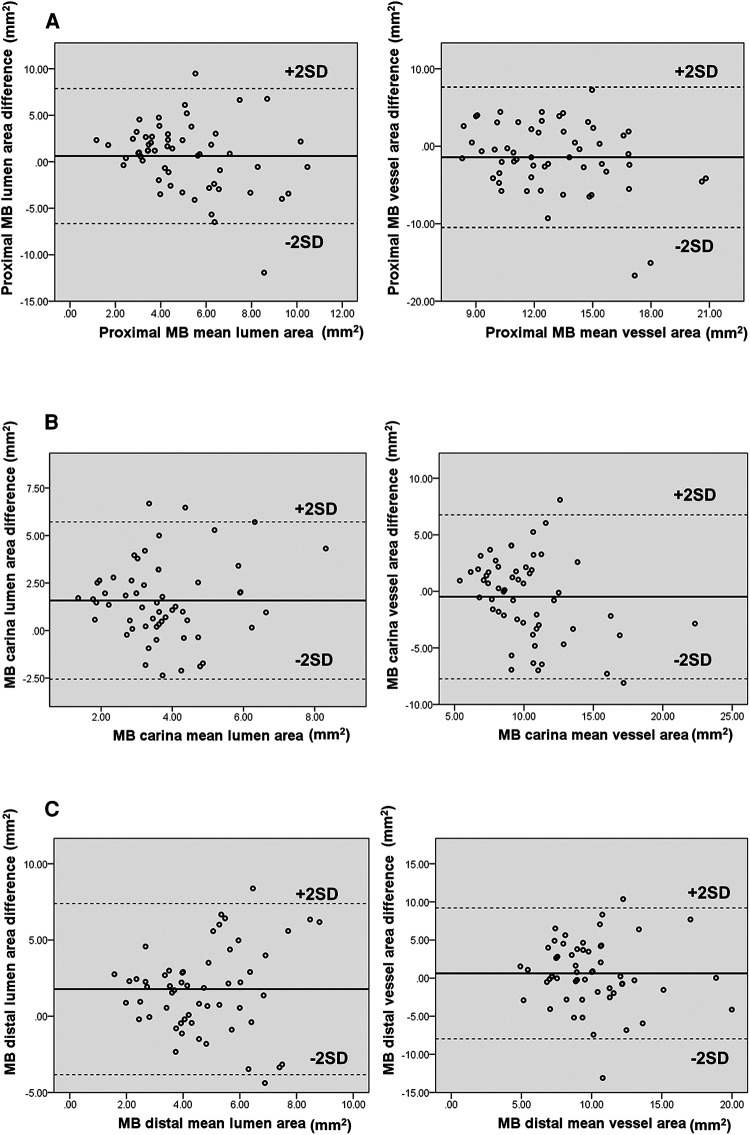
(**A–C**) Diagram displays Bland–Altman plots of lumen and vessel area measurements’ differences in mm^2^ using CTCA versus IVUS at proximal (**A**), carina (**B**), and distal segment (**C**) of MB bifurcation. The diagram shows moderate correlation with wide limits of agreement.

The differences in vessel and lumen area measurements between CTCA and IVUS of the MB were pronounced regarding the lumen area at the MB carina and distal segment, together with the MLD segment. On the other hand, the significant differences in the vessel area were shown on proximal MB and MLD segments of bifurcation (Table 4). Bland–Altman analysis showed a discrepancy in measuring mainly the lumen area between CTCA and IVUS in proximal MB [lumen 5.10, 95% CI (4.53–5.68) mm^2^, *p* < 0.001; vessel −1.42, 95% CI (−2.63 to −0.21) mm^2^, *p* = 0.023), carina MB [lumen 3.74, 95% CI (3.37–4.10) mm^2^, *p* < 0.001; vessel −0.48, 95% CI (−1.45 to 0.48) mm^2^, *p* = 0.322], and distal MB [lumen 4.72, 95% CI (4.27–5.18) mm^2^, *p* < 0.001; vessel 0.62, 95% CI (−0.53 to 1.77) mm^2^, *p* = 0.283]. The plot demonstrated that most of the measurements were within the limits of 2 SD (Figure 4A–C).

**Table 4:** Mean values and differences in the lumen and vessel area of the bifurcation's MB measured using CTCA and IVUS.

Variable	CTCA	IVUS	Difference	*p*-Value
Proximal segment				
Lumen area (mm^2^)	4.80 ± 3.19	5.41 ± 2.41	−0.61 ± 3.63	0.214
Vessel area (mm^2^)	13.70 ± 4.25	12.28 ± 3.11	1.42 ± 4.53	0.023
Carina segment				
Lumen area (mm^2^)	2.95 ± 1.63	4.53 ± 1.79	−1.58 ± 2.07	<0.001
Vessel area (mm^2^)	10.49 ± 4.11	10.01 ± 2.97	0.48 ± 3.62	0.322
Distal segment				
Lumen area (mm^2^)	3.87 ± 2.06	5.59 ± 2.34	−1.72 ± 2.81	<0.001
Vessel area (mm^2^)	9.62 ± 3.85	10.24 ± 3.59	−0.62 ± 4.29	0.283
MLD segment				
Lumen area (mm^2^)	0.97 ± 0.49	3.19 ± 0.82	−2.22 ± 0.95	<0.001
Vessel area (mm^2^)	13.78 ± 5.00	10.75 ± 2.84	3.03 ± 4.89	<0.001

**Figure 5:**
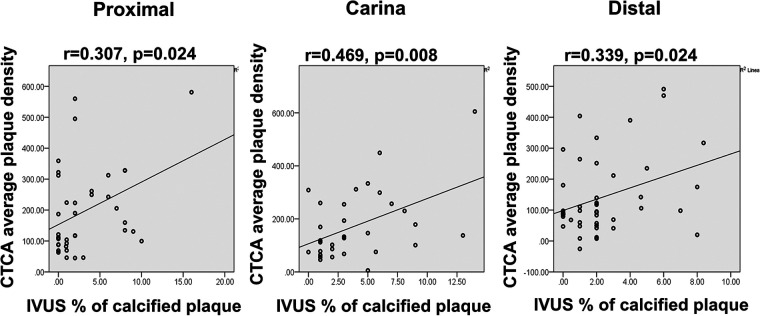
Diagram displays the correlation scatter plot between the CTCA average plaque densities (y-axis) and percentage of calcified plaque on IVUS tissue analysis (x-axis) in the proximal, carina, and distal segment of MB bifurcation (polygon of confluence). *p*, level of significance; *r*, correlation coefficient.

When comparing tissue characteristics of the MB as assessed by CTCA and IVUS, most of the plaques were classified as fibrotic on CTCA which is in concordance with fibrous tissue dominance on IVUS virtual histology (VH) (Table 5). A significant correlation was noted between MB average plaque density in HU on CTCA with the percentage of calcified atherosclerotic plaque on IVUS tissue characterization at every level of “polygon of confluence” (Figure 5).

**Table 5:** Bifurcation's tissue characterization assessed by CTCA and IVUS.

Variable	Proximal segment	Carina segment	Distal segment	MLD segment
CTCA				
Average plaque density (HU)	183 ± 188	156 ± 127	138 ± 142	160 ± 202
Plaque classification^a^	F	F	F	F
IVUS				
Fibrous plaque (%)	66 ± 13	61 ± 13	61 ± 14	53 ± 13
Lipid plaque (%)	10 ± 4	11 ± 4	12 ± 4	11 ± 4
Necrotic plaque (%)	22 ± 11	25 ± 11	25 ± 11	32 ± 11
Calcified plaque (%)	2 ± 2	3 ± 3	2 ± 3	4 ± 2

^a^ Plaque classification based on density lipid (L) (−30–75 HU), fibro-lipid (FL) (76–130 HU), fibrous (F) (131–350 HU), and calcified (C) (more than 351 HU).

## 4. Discussion

We conducted the study in which we evaluated the use of CTCA in a complex, bifurcation lesion that had advanced atherosclerotic disease with significant plaques in both MB and SB. Our study demonstrated that in “true” non-left main bifurcations plaques located on the carina side of MB and SB have lower densities compared with plaques located on the lateral walls of the MB and SB on CTCA. Importantly, atherosclerotic plaques that “encroach” the entire circumference of the proximal segment of the MB were independently associated with SB flow compromise after “provisional” stenting of the MB and POT. Fair correlation existed between CTCA and IVUS in measuring vessel diameters and when comparing plaque density on CTCA and percentage of calcified plaque on IVUS VH.

Atherosclerotic plaques in coronary artery bifurcations tend to develop in the areas of low endothelial shear stress (ESS), at the lateral walls of bifurcations, where the endothelium is stimulated by local growth factors and relatively slow, laminar blood flow. On the other hand, in the areas with high ESS located usually at the flow divider of the bifurcation, plaque rarely develops and has different characteristics (17, 18). In a study of 65 bifurcations, van der Giessen et al. demonstrated that atherosclerotic plaques mostly developed in the areas of low wall shear stress and that calcium-rich plaques are situated in the lateral portions (19). It was also demonstrated by Eshtehardi et al. that areas with high ESS show a negative correlation with specific plaque characteristics (necrotic core and dense calcium) (20).

Our series of bifurcations comply with an observed pattern, and plaques at the carina side of MB and SB were less frequently found compared with plaques at the lateral side. We found that plaques at the lateral side of the bifurcation contain more dense plaques compared with the plaques on the flow divider side.

The SB compromise, defined as a decrease in coronary flow below TIMI 3, occurred in almost one-fifth of our patients. The presence of the plaque on the carina side in the SB was associated with SB compromise in univariate analysis, but this relationship was not confirmed in multivariable model. In our study, only circumferential plaque in the proximal segment of the MB was independently associated with SB compromise. This could be the consequence of the large plaque volume in the proximal MB being pushed toward the SB ostium by stent implantation which, when added to ostial stenosis of the SB, might result in a critical stenosis or occlusion of the SB and decreased TIMI flow (snow plough effect). This finding corroborates with large registry of bifurcation PCI which demonstrated that significant stenosis of proximal MB is associated with SB compromise (21, 22). Moreover, an optical coherence tomography study by Kini et al. demonstrated that patients with SB occlusion had greater diameter stenosis of proximal MB, although MB stenosis was not an independent predictor of SB compromise, as opposed to the lipid content of the proximal MB plaque (23). Recently developed prediction scores that use CT often depict low attenuation and calcified plaque of proximal MB as significant contributors to SB compromise (24, 25). The diameter ratio between MB and SB on CTCA did not show relevant association with SB compromise in our study, contrary to the validated RESOLVE score that include the MB/SB ratio as an important predictor of SB decreased TIMI flow. The reason for this may be attributed to the complexity of bifurcations included in our study leading to very high RESOLVE scores in which the MB/SB ratio would have relatively small contribution especially if we know that the average MB/SB ratio in our study was 1.3 ± 0.3. This value would only add two points to the RESOLVE score which, in our group of patients, would be more affected by diameter stenoses of MB and SB, bifurcation angle, and plaque distribution (13, 26).

Our study included patients with large, advanced plaque burden of the bifurcation, in whom we were unable to perform volumetric plaque analysis to evaluate the possible effect of “plaque shift” on the SB fate. This limitation of our study in evaluating plaque behavior attributed to MB stent implantation must be noted. A precise automated volumetric plaque analysis on CTCA would add valuable information about plaque burden and its behavior after stent implantation.

Previous studies demonstrated a good correlation between CTCA and IVUS in quantifying intermediate coronary atherosclerotic lesions with the notion that CTCA overestimates the lumen area (26, 27).

In our study measurements of the vessel area were similar at all levels of bifurcation, which was not true for lumen measurement, especially at the MLD point in the MB. In concert with previous studies, in our population CTCA overestimated the lumen area of the bifurcation. The average plaque density at the MLD level was 144 ± 138 HU with a maximum density of 541 HU. The calcium density on CTCA approaches the density of contrast fluid and could impede detection of lumen and vessel borders leading to the evidence that inaccurate CTCA vessel and plaque quantitative measurements are associated with greater calcified plaque volume, increased percentage of calcified plaque, and lumen diameter smaller than 2.8 mm (28–30). It should be noted that we did not perform calcium scoring according to Agatston score in order to reduce radiation exposure, and we excluded patients with type III and IV calcified lesions on CT. These lesions could interfere with the vessel analysis on CT and influence the correlation between measurements performed on CTCA and IVUS (31). Since our trial included vessels with relatively large diameters (Table 2), the accuracy of CTCA has been preserved, because it has been previously shown that small vessels and extensive calcification interfere with the accuracy of CT evaluation (32).

We found a moderate correlation for average plaque density on CTCA and percentage of calcified plaque on IVUS tissue characterization in all segments of the bifurcation's MB. The reason for this could be sought in the observation that IVUS VH software often misclassifies the zone of acoustic shadow behind the calcified plaque and that spatial resolution of the CT scanner that we used may not be able to reliably discern fibrous and lipid plaque, as IVUS can do (33, 34). If we combine shortcomings of CT coronary angiography regarding differentiation of fibrous and lipidic plaques and the effects of calcified plaque on IVUS VH that can be the reason for relatively moderate correlation regarding between these modalities regarding plaque identification. The IVUS VH software itself, due to these issues, has been abandoned in the new versions of IVUS proprietary software.

### 4.1. Limitations of the study

It should be noted that we performed semiautomated anatomical co-registration between CTCA and IVUS which can lead to a certain bias in measurements and interpretation of the results. Automated software that can perform precise co-registration would lead to increased accuracy of the measurements and better correlation between imaging modalities (35). When evaluating circumference cross-section of the MB and SB, we were not able to perform plaque thickness quantitative analysis that could have influence on carina shift and potential SB compromise.

### 4.2. Impact on daily practice

Thorough plaque identification and quantification using CTCA could give a better insight for an interventionalist about the distribution and extent of atherosclerotic disease in complex coronary bifurcation. This non-invasively acquired information can cause a treating physician to anticipate difficulties in PCI and to decide to change the initial strategy toward a more complex one and to make a better choice of stent diameter and length. All this can improve the use of resources and minimize procedural risks.

## 5. Conclusion

Detection and characterization of atherosclerotic plaque by CT coronary angiography in non-left main “true” coronary bifurcations can provide useful information about bifurcation anatomy and plaque distribution that can predict outcomes after provisional stenting, thus guiding the interventional strategy to bifurcation PCI. It also gives comparable results to IVUS regarding vessel sizing and plaque characterization.

## Data Availability

The original contributions presented in the study are included in the article/**Supplementary Material**, further inquiries can be directed to the corresponding author.
