# Supplementary material for: Multislice computerized tomography coronary angiography can be a comparable tool to intravascular ultrasound in evaluating “true” coronary artery bifurcations
Source: Front Cardiovasc Med. 2023 Nov 6;10:1292517. doi: 10.3389/fcvm.2023.1292517 (PMC10657987; doi:10.3389/fcvm.2023.1292517)
Supplement: Supplementary file 1 [file Datasheet1.docx]

Supplementary Material

# Supplementary Data

- 1. **Supplementary Data 1 –** CTCA procedure

Patients underwent CTCA on Aquilion CXL 128 slice CT scanner (Toshiba Medical Systems Europe, Zoetermeer, The Netherlands) using predefined protocol. The procedure included CT coronary angiography using Ultravist 370 contrast agent (iopromide concentration 370 mg/ml, Bayer Health Care, Germany). The angiograms were analyzed using dedicated software Vital Vitrea Advanced 6.2 (Vital Images, Minnetonka, Minnesota, USA). A detailed description of the procedure is shown in Supplementary Data 1. The bifurcation lesion analysis included the measurement of proximal and distal reference vessel diameters (RVD) of the MB and RVD of the SB at the point least affected by atherosclerosis up to 10 mm from the bifurcation’s stenosis, MB vessel and lumen diameters 5 mm proximal to carina, at carina level and 5 mm distal to carina (polygon of confluence), and at the site of MB’s greatest luminal narrowing – minimal lumen diameter (MLD). The same analysis was done at the SB ostium. Also, the angles between MB and SB were measured. Plaque analysis was performed in the longitudinal cross-section of the bifurcation at lateral and carina side at every previously mentioned location in the MB and SB. Also, circumferential cross-section plaque analysis was performed to locate the presence of plaque on every point of the vessel circumference - lateral, carina, myocardial and pericardial side. The plaque analysis in the longitudinal cross-section included determination of type of the tissue based on measured density in Hounsfield units (HU), so that plaque with density up to 75 HU was marked as lipid, 76 – 130 HU as fibro-lipid, 131 – 350 HU as fibrous, and above 351 HU as calcified plaque (8).

- 1. **Supplementary Data 2 –** IVUS procedure

After placing coronary guidewires in MB and SB, MB IVUS was performed using Opticross 40MHz IVUS catheter (Boston Scientific Corporation/Scimed, Natick, Massachusetts, US). The withdrawal of the catheter was performed using automated motorized pullback at a constant speed of 0.5mm/s.

The acquired data were stored and transffered for an off-line analysis using commercialy available software QIvus 3.0 (Medis Medical Images Software, Leiden, The Netherlands). The lumen and the media-adventitia borders were defined by automatic contour detection software with manual editing at the frames of interest.

Tissue characterization was done using iMAP software (Boston Scientific Corporation/Scimed, Natick, Massachusetts, US) which uses color codes to describe the composition of the atherosclerotic plaque so that fibrous tissue is coded green, lipid as yellow, necrotic as purple and calcified plaque as light blue. The results were expressed as percentage of total segmental plaque for each tissue.

The anatomical co-registration between two imaging modalities was performed by identifying carina on IVUS longitudinal and cross-section as a last frame where entire circumference of MB and SB can be visualized before confluence. The points 5mm proximal and distal to carina in "polygon of confluence“ were then identified. On longitudinal IVUS recording the distance of MLD point from bifurcation’s carina was calculated. The carina was identified on CTCA cross-section using same principle as in IVUS recording, then other three points of interest were detected using distances obtained in IVUS longitudinal section (Figure 1).

- 1. **Supplementary Data 3 –** PCI procedure

Initial strategy for PCI in all cases was "provisional“ stenting of the MB. The choice of vascular access, guiding catheters and coronary wires were left to the operators' discretion. After MB predilation, a second generation drug eluting stent (DES) was placed across the SB, with diameter chosen according to visual estimate of distal MB in accordance with Murray's law(9). After proximal optimization technique (POT) using short noncompliant balloon catheter, with high-pressure inflation and intracoronary nitroglycerin administration, coronary angiograms in two orthogonal projections were done. If SB coronary blood flow was less than thrombolysis in myocardial infarction (TIMI) 3, procedure was continued with guidewire exchange, preferably using a third guidewire in an attempt to pass through the distal strut in the SB. If after angioplasty of the SB ostium and/or kissing balloon inflation the SB TIMI flow was less than 3 and/or there was an ostial dissection, another second generation DES was implanted in the SB using the technique chosen by the operator.

After the procedure, patients who had an uneventful PCI stayed in the hospital for 24 h. Blood samples for Troponin I, CK and CK-MB were collected at 12 and 24 h after the procedure. Complications of the interventions were documented in the patients’ study file. Patients were seen during scheduled visits in the office at 1, 6 and 12 months after the procedure.

**
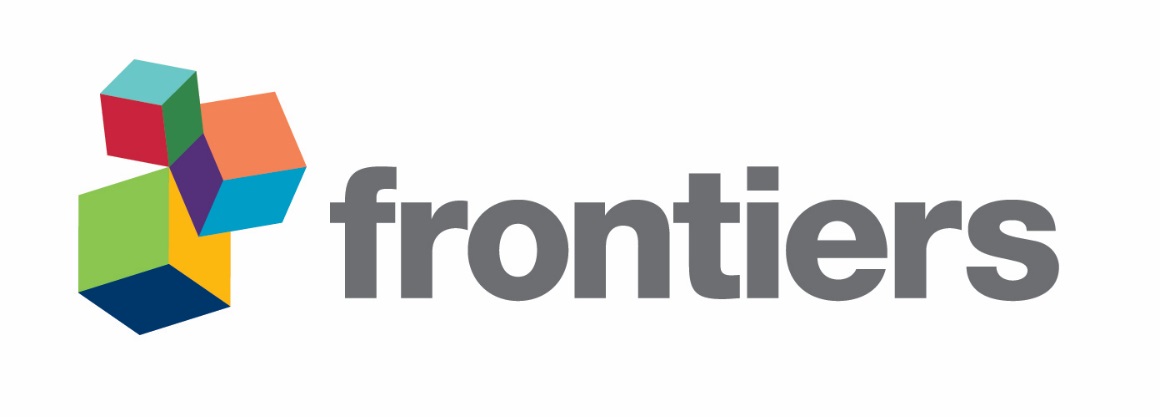
**
